# Case Report of Untreated Pediatric Femoral Neck Fracture with Osteopenia

**DOI:** 10.21980/J8S92K

**Published:** 2020-04-15

**Authors:** Sha Yan

**Affiliations:** * Donald and Barbara Zucker School of Medicine at Hofstra/Northwell, Southside Hospital, Department of Emergency Medicine, Bay Shore, NY

## Abstract

Pediatric hip fractures are rare. This type of injury often requires surgery within 24 hours to prevent complications such as avascular necrosis, bone non-union, and premature physeal closure. Moreover, chronic disuse from untreated fracture causes severe osteopenia. Untreated pediatric hip fracture is uncommon in the developing world due to access to surgical care. Here, we report a pediatric patient who traveled from Honduras with untreated right femoral neck fracture. The delay in her initial surgical intervention left her with chronic non-union of her right hip joint and significant osteopenia of her right leg secondary to chronic disuse. This patient required a total right hip arthroplasty rather than the conventional internal fixation. It is crucial for emergency physicians to identify the causes of pediatric hip pain. Early detection of these pathologies allows for early orthopedics involvement and surgical intervention to avoid long-term complications.

**Topics:**

Pediatric orthopedic, femoral neck fracture, osteopenia.

[Fig f1-jetem-5-2-v1]

**Figure f1-jetem-5-2-v1:**
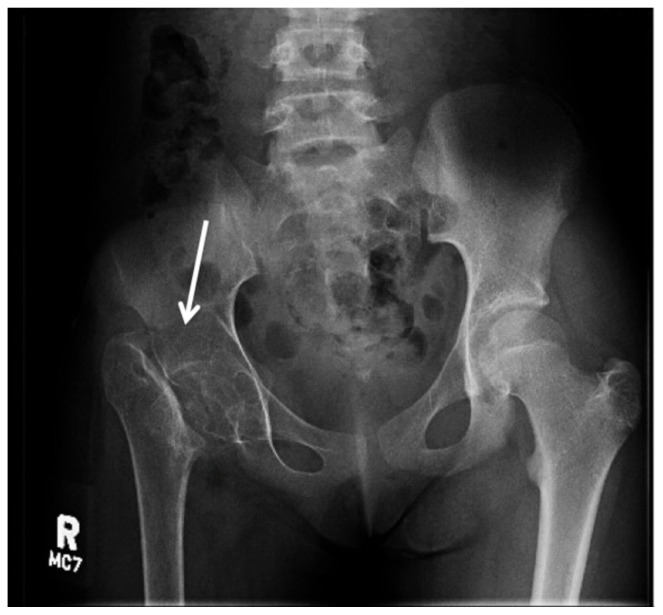

[Fig f2-jetem-5-2-v1]

**Figure f2-jetem-5-2-v1:**
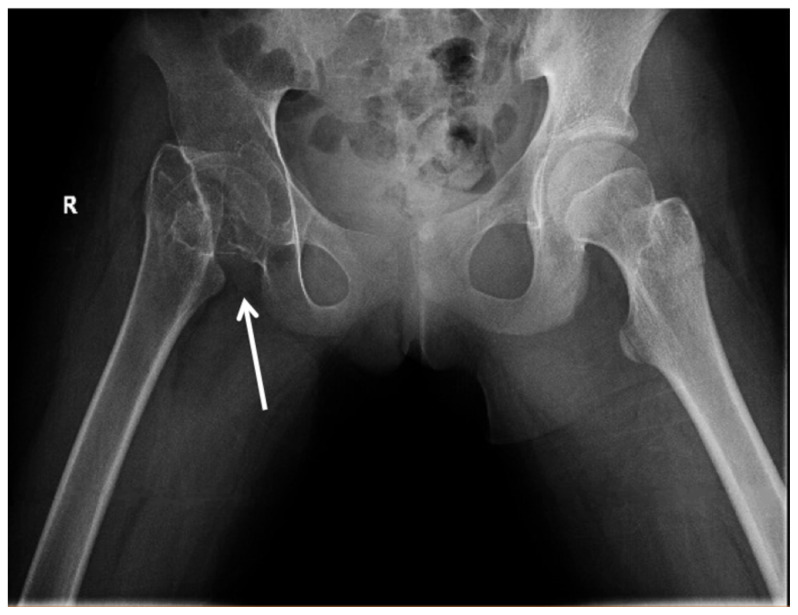

## Introduction

Pediatric femoral neck fractures account for less than 1% of all fractures in children.^1^–^4^ Typically, this is a result of a high-energy trauma or fall from significant height.^1^–^4^ This type of injury requires urgent surgery to prevent long term complications such as avascular necrosis, coxa vara, chondrolysis, premature physeal closure, and infection.^1^–^4^

## Presenting concerns and clinical findings

A 13-year-old female without past medical conditions presented with localized right hip pain of two years duration. Her father reported that she fell from a tree two years ago in Honduras and fractured her right hip. Surgery was offered but her father reported that he was not able to afford surgery. Hence, she has been dependent on crutches for ambulation because she has been unable to bear weight on her right leg. Patient and her father flew to the United States seeking medical treatment. On review of systems, she reports chronic right hip pain, no fever, and no numbness. On exam, she had shortened right leg with external rotation, right hip tenderness, limited range of motion up to 30 degrees in flexion, full range of motion at the right knee and ankle, sensations are intact, < 2 seconds capillary refills, and 2+ peripheral pulse bilaterally. No physical abnormality of the left leg was found. No laboratory blood tests were sent.

## Significant findings

On her right hip radiograph, the patient was found to have a right femoral neck fracture with superior displacement of the intertrochanteric portion of the right femur. Moreover, the radiograph demonstrated diffuse osteopenia of the right hip and femur from chronic disuse as characterized by the increased radiolucency of the cortical bones compared to the left side.

## Patient course

During the ED course, a pediatric orthopedist was consulted over the phone. Given the chronicity and the diffuse osteopenia, emergent surgery was not indicated at this time. The orthopedic consultant requested additional images to further evaluate the condition. A CT scan of the right hip showed chronic mildly displaced right subcapital femoral neck fracture. Radiography of her right lower extremity showed diffuse osteopenia but no additional fractures. Patient was discharged from the ED with outpatient follow up. Eight months later, the patient had a total right hip arthroplasty with an adult orthopedic team at a children’s hospital. Her surgical course was uncomplicated. She was discharged with home physical therapy, touch down weight bearing of the right leg with crutches, and strict posterior hip precautions, which included no crossing of the legs and no flexing above 95 degrees. During her post-operative follow up, she was still walking with a stiff gait and needing assistance with crutches, but it was noted by her orthopedic surgeon that she had improved range of motion with straight leg raise to 60 degrees and flexion to 90 degrees. There were no signs of surgical infections, and she no longer required pain medication. She was instructed to continue with outpatient physical therapy; however, there are no further follow-ups within our hospital system so her ultimate outcome is unclear.

## Discussion

Pediatric hip fracture is rare, accounting for < 1% of all pediatric fractures.^1^–^4^ They are frequently associated with high-energy trauma such as falling from a significant height, as in the case of this patient, or from high-speed motor vehicle accidents.^1^–^4^ Since the femoral head is exclusively supplied by the lateral epiphyseal artery with no anastomosis between the epiphysis and femoral neck, injury to this area can lead to complications such as avascular necrosis (AVN), bone non-union, coxa vara, chondrolysis, premature physeal closure, and infection.^2^ AVN is the most common and serious complication, and the rate of AVN varies significantly between 0% – 92% among different reports.^1^ Surgical reduction of femoral neck fracture with either closed or open internal fixation realign the bone to preserve vascular supplies and prevent the progression of AVN and non-union. 3 Study shows that patients with delayed surgical intervention of >24 hours were 4.5 times more likely to develop AVN compared to early intervention of < 24 hours.^4^ Hence early identification and intervention is crucial.

Replacement arthroplasty is not the first line of treatment for femoral neck fracture. Bone stalks should be preserved if possible.^3^ However, when AVN progresses and causes subchondral collapse, the definitive treatment is a total replacement arthroplasty.^3^ Complication of arthroplasty in the young includes dislocation and acetabular erosion that often require revision at some point during their lifetime.^3^

While delayed surgical intervention affects the vascular and osteo development around the hip joint itself, the prolonged periods of immobility cause remodeling and decreased bone density of the whole extremity. The decrease in bone mass, in turn, increases the vulnerability to new fractures.^5^ Bone development depends on mechanical loading. It is particularly important in adolescence during Tanner stage II–IV when the bone mass is most responsive. Some studies suggest high impact activity even before the initiation of puberty has a positive bone growth later in development.^6^ As a result of this patient’s chronic fracture, her x-ray showed significant osteopenia from her inability to bear weight.

Untreated pediatric femoral neck fracture is nearly unheard of in developing countries. Here we see a rare image of an untreated pediatric femoral neck fracture with osteopenia. Complication of untreated femoral neck fracture is debilitating and requires surgical management. It is crucial for emergency physicians to identify potential etiology of pediatric hip pain such as septic arthritis, Perthes disease, slipped capital femoral epiphysis, apophyseal avulsion fractures, and, as in this patient, traumatic femoral neck fracture. Early detection of these conditions allows for early orthopedics involvement and surgical intervention to avoid long-term complication.

## Supplementary Information








