# Supplementary figures and images for: Case Report of Untreated Pediatric Femoral Neck Fracture with Osteopenia
Source: J Educ Teach Emerg Med. 2020 Apr 15;5(2):V1–3. doi: 10.21980/J8S92K (PMC10332562; doi:10.21980/J8S92K)

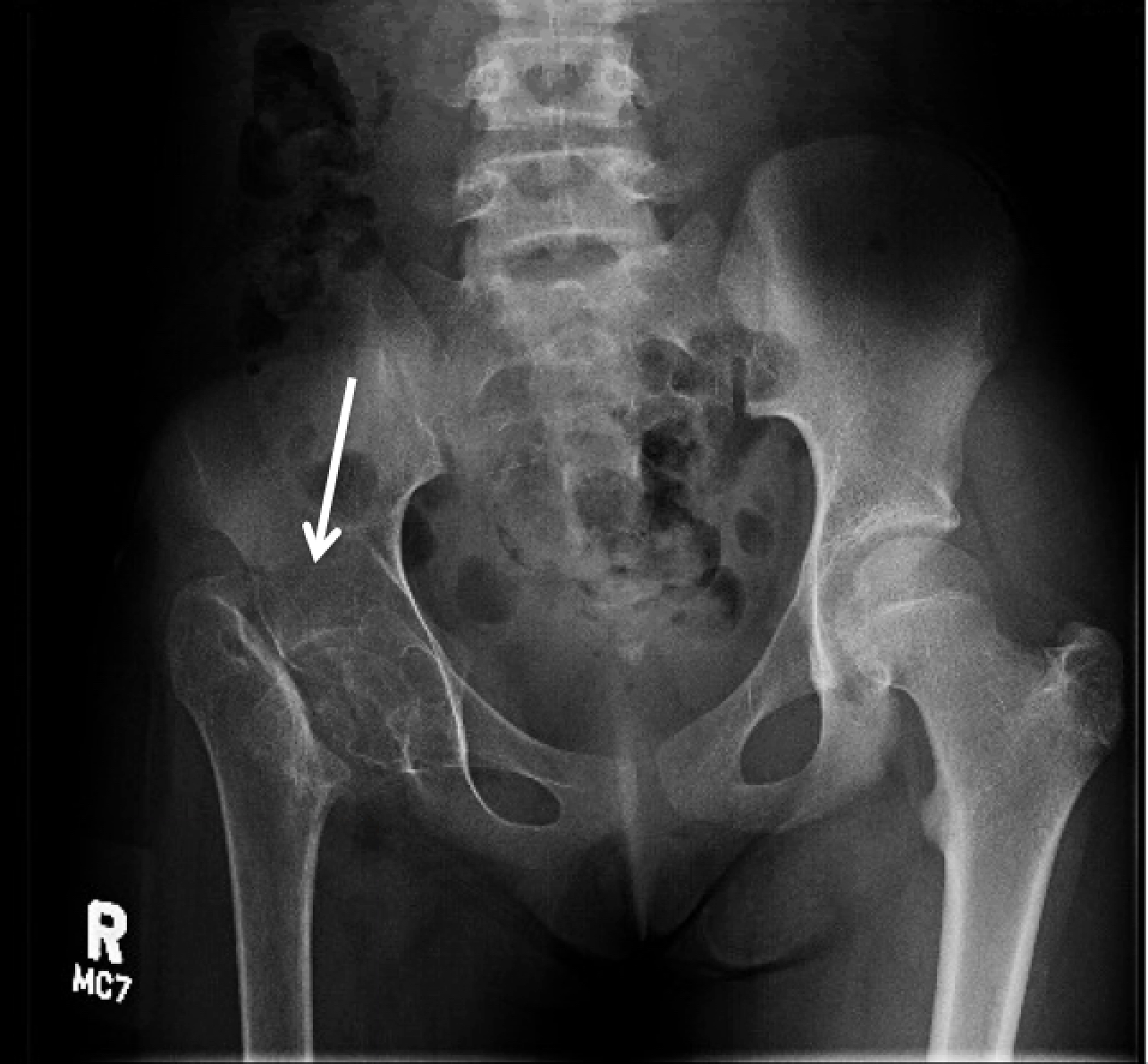

Supplement: Supplementary file 1 [file jetem-5-2-v1-supp1.jpeg]

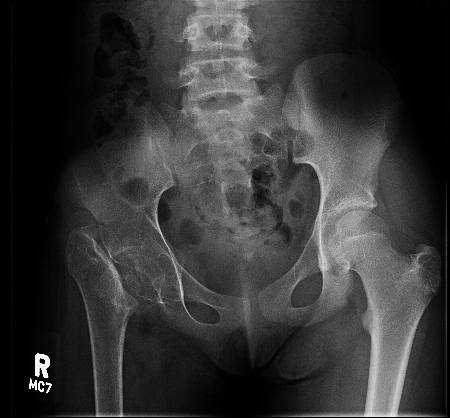

Supplement: Supplementary file 2 [file jetem-5-2-v1-supp2.jpeg]

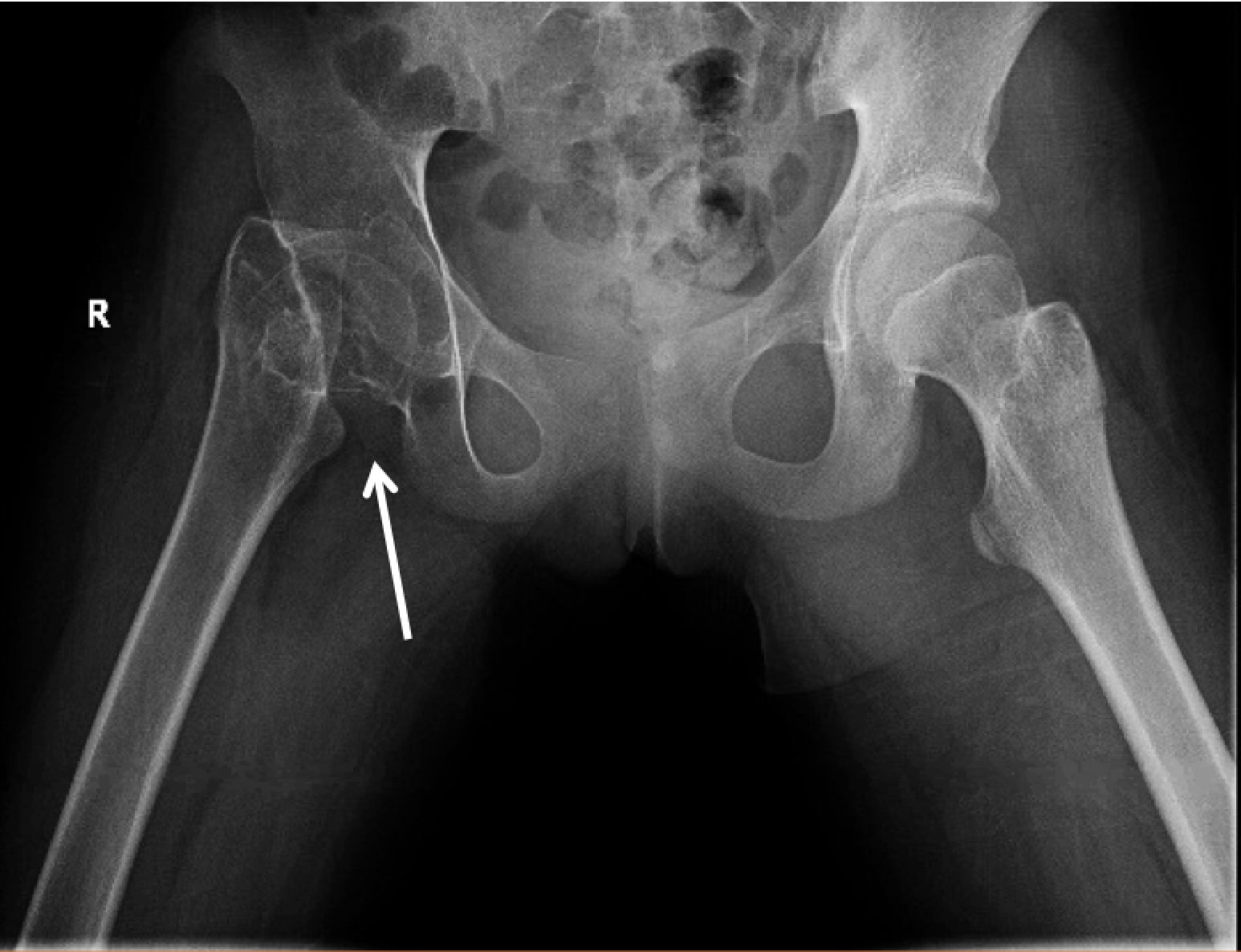

Supplement: Supplementary file 3 [file jetem-5-2-v1-supp3.jpeg]

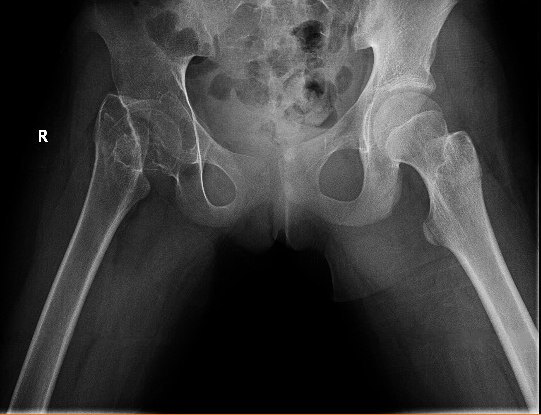

Supplement: Supplementary file 4 [file jetem-5-2-v1-supp4.jpeg]
